# Changes in high school students’ interest in medical school following a physiology-based medical science competition: a questionnaire-based study

**DOI:** 10.1186/s12909-026-10081-5

**Published:** 2026-08-03

**Authors:** Supawat Tiravanitkul, Chantacha Sitticharoon, Issarawan Keadkraichaiwat, Phakhathorn Piandecha, Arisra Aubdullakacim, Aukapatra Jaruchaiyong, Inpreeya Inpaen

**Affiliations:** Department of Physiology, Faculty of Medicine Siriraj Hospital, Mahidol University, 2 Wanglang Rd., Siriraj, Bangkok Noi, 10700 Bangkok, Thailand

**Keywords:** Medical school, Academic competition, Physiology, High school student, Motivation

## Abstract

**Background:**

The 1^st^ Siriraj Medical Sciences Academic Competition (SiMSAC), themed ‘Physiology,’ integrated gamification and experience-enhancing activities to motivate high school students to pursue medical studies. However, evidence on whether such competitions are associated with students’ interest in medical school remains limited. This study aimed to evaluate changes in high school students’ interest in medical school (Med-Interest), interest in the organizing institution (SI-Interest), and self-assessed physiology knowledge following participation in a physiology-based competition, and to examine correlations with high school subject preferences and satisfaction with competition activities.

**Methods:**

This retrospective pre-post within-subject questionnaire-based study used census sampling of respondents (99.3%, 569/573). Participants were students in grades 9–12 from 191 schools across Thailand. Data were collected after the competition using a self-developed questionnaire comprising quantitative rating scales and optional open-ended questions on participants’ impressions of the competition and suggestions. Participants retrospectively rated Med-Interest and SI-Interest before and after the competition, and self-assessed physiology knowledge before preparation, during preparation, and after the competition. The evaluation also incorporated findings from the organizing committee’s departmental After Action Review (AAR).

**Results:**

Mean (95% CI) Med-Interest increased from 4.0 (3.9–4.1) before the competition to 4.4 (4.3–4.5) after the competition, and SI-Interest increased from 3.7 (3.6–3.8) to 4.2 (4.1–4.3) (all *P*<.001), corresponding to relative increases of 13.8% (11.4%–16.4%) (Cohen’s d = 0.562) and 23.1% (19.3%–27.2%) (Cohen’s d = 0.675), respectively. Self-assessed physiology knowledge increased from 3.2 (3.1–3.3) before preparation to 3.8 (3.7–3.8) during preparation and 4.2 (4.1–4.2) after the competition (*P*<.001). Higher preferences for Biology, Chemistry, and Physics were associated with higher Med-Interest and SI-Interest (*P*<.05). Post-competition Med-Interest and SI-Interest were positively but weakly correlated with satisfaction with competition activities and subject preferences (ρ = 0.123–0.321 and 0.186–0.340; all *P*<.01).

**Conclusions:**

Participation in a physiology-based academic competition was associated with increased interest in medical school, with a greater increase in interest in the organizing institution, along with improved self-assessed knowledge. These findings were further supported by participants’ optional open-ended responses and the organizing committee’s AAR, highlighting the value of integrating academic challenge, gamification, and experiential learning in pre-medical outreach and student engagement.

**Supplementary Information:**

The online version contains supplementary material available at https://doi.org/10.1186/s12909-026-10081-5.

## Introduction

Academic competitions can bring excitement to participants by engaging them as part of a team and offering opportunities to learn new things, meet new people, experience enjoyment, compete with peers from other schools, and gain rewards or recognition [1].

Academic competitions may incorporate various test formats, including multiple-choice questions (MCQs) and open-ended questions (OEQs). Through gamification, which involves the utilization of game-design elements in non-game contexts [2] such as scoring systems, leaderboards, rewards, and varying levels of difficulty [3–5], these approaches can influence individuals’ behavior and enhance both intrinsic motivation, such as a sense of achievement, and extrinsic motivation, such as social acceptance, earning points, and receiving rewards [3]. In addition, participating in science-related activities can potentially influence students’ motivation to choose a science program in the future [6], including their decision to pursue medical school [7, 8].

Regarding motivation to choose future studies, the Expectancy-Value Theory suggests that expectancy of success and task value can impact one’s persistence, performance, and choice concerning the task [9]. These constructs—expectancy of success and task value—are determined by motivational beliefs, including goals, perceived task difficulty, self-concept, and affective memories, which are shaped by past events or social influences through personal perspectives or perceptions [9, 10]. Self-concept refers to an individual’s general perception of their own competence within a particular domain, such as academic ability [9]. Within this framework, learners’ personal interpretations of their experiences and environment may shape how social influences are translated into motivational beliefs.

To influence students’ motivation to study medicine at our medical school, the 1^st^ Siriraj Medical Sciences Academic Competition (SiMSAC), themed ‘Physiology,’ was organized to promote our medical school nationwide and encourage students to choose our program as their preferred medical school by using physiology knowledge from the high school curriculum to build a bridge between high schools and the medical school. Although medical school admission is highly competitive, our aim was not simply to increase general interest in pursuing medicine, but also to position our institution as a medical school of choice. The competition was therefore designed to introduce students to what studying medicine may entail, including the learning environment, curriculum, and educational experiences at the Faculty of Medicine Siriraj Hospital (SI). By combining academic competition with experiential activities, we sought to increase awareness of our institution and help students make more informed decisions about applying to our medical school.

To our knowledge, no previous study has examined the impact of participation in a medical science competition on students’ interest in medical studies. This study aimed to investigate the impact of a physiology-based medical science competition on high school students’ interest in medical school (Med-Interest), interest in our institution (SI-Interest), and self-assessed physiology knowledge. Subgroup analyses based on participants’ preference levels for high school subjects were performed to explore whether students with different subject preferences showed different patterns of Med-Interest, SI-Interest, and self-assessed physiology knowledge. In addition, correlations between their Med-Interest and SI-Interest and their preference levels for high school subjects, self-assessed physiology knowledge, and satisfaction with academic and experience-enhancing activities were examined. The findings of this study may provide practical insights for designing and improving medical science outreach and pre-medical enrichment activities to support students’ motivation toward medical studies.

## Methods

### Competition participants and activities

Participation in SiMSAC, held on February 18–19, 2023, was open to high school students in grades 9–12 nationwide. The invitation specified enrolment at the high school level as the only eligibility criterion, and each school was invited to nominate 1 representative team consisting of 3 students, with team member selection determined by the schools. Prior interest in medicine or intention to pursue a medical career was not an eligibility criterion. A total of 191 schools (191 teams; *N* = 573 students) participated in the competition, representing all regions of Thailand, including the Northern, Central, Northeastern, Eastern, Western, and Southern regions.

Competition activities comprised academic and experience-enhancing components. The competition was primarily designed as an academic outreach activity to promote medical education and provide learning experiences related to physiology, rather than to formally evaluate or compare academic ability among participants across grade levels. Therefore, competition scores and performance outcomes were not included as study variables in the present analysis.

For the academic component, the competition consisted of 4 rounds:

1. MCQ round: A total of 191 teams, each comprising 3 members, participated in this round (*N* = 573). The examination was conducted as a computer-based test using the same system routinely used for medical student assessments, with a total duration of 90 min. The top 30 teams advanced to the Qualifying round.
2. Qualifying round: A total of 30 teams, divided into 5 rounds with 6 teams per round, competed by answering 9 OEQs in writing on a whiteboard. The top 2 teams from each round, totaling 10 teams, advanced to the Semi-finals.
3. Semi-finals: A total of 10 teams, divided into 2 rounds with 5 teams per round, competed with oral OEQs, each consisting of 10 questions. The top 2 teams from each round, totaling 4 teams, advanced to the Final round.
4. Final round: A total of 4 teams competed in 12 oral OEQs.

A detailed description of the competition format and activities is provided in Supplementary Appendix 1.

Awards and incentives were provided to recognize participants’ performance in the competition. Based on the MCQ round results, the top 10 individual participants with the highest scores each received an individual award of 1,000 THB. All participants received a certificate of participation. Team prizes were awarded to the top 4 teams, with the highest-scoring team receiving first prize (5,000 THB and a certificate), followed by second prize (3,000 THB and a certificate), third prize (2,000 THB and a certificate), and 1 honorable mention for the fourth-ranked team (1,000 THB and a certificate).

Experience-enhancing activities consisted of:

1. SI talk: An interactive talk show-style session involving the Assistant Dean for Undergraduate Education of the Faculty of Medicine Siriraj Hospital and medical students, providing information on the Doctor of Medicine (MD) program and the learning experience at the faculty. The session lasted approximately 1 h and was held in a large lecture hall used for medical student teaching, with a seating capacity of approximately 400. The session utilized a three-screen projection system and interactive audience engagement using Mentimeter to simulate a real medical school learning environment.
2. Gross anatomy laboratory visit: A variety of specimens were displayed in the gross anatomy laboratory, and participants were guided by medical students to experience the actual learning environment used for anatomy practical sessions. Participants entered the laboratory in small groups for approximately 10 min per group. The laboratory used for the activity was the same gross anatomy laboratory where medical students conduct their anatomy practical sessions.
3. Museum visit: Participants were provided with complimentary access tickets to all museums in Siriraj Hospital, including the Ellis Pathological Museum, the Parasitology Museum, the Songkran Niyomsane Forensic Medicine Museum, the Congdon Anatomical Museum, and the Siriraj Bimuksthan Museum. They were free to visit the museums independently during the allocated activity period.
4. Performance activity: An activity designed to build relationships among participants, including performances by medical students and participants. Participants were invited to volunteer for stage performances, followed by the announcement of the 30 teams advancing to the qualifying round. The activity lasted approximately 3 h and was held in the same 400-seat lecture hall used for the SI talk session.

Although only the qualifying teams advanced to subsequent competition rounds, all participants remained eligible to participate in the experience-enhancing activities throughout the event. Participants who did not advance were also able to observe the Qualifying, Semi-final, and Final rounds either onsite or through live online streaming, with recordings available for later viewing. Consequently, all participants had the opportunity to experience the overall competition program before completing the post-competition questionnaire.

### Questionnaire

After the competition, participants were invited to complete a single online post-competition questionnaire in Thai via Google Forms, distributed by email, which was administered immediately after the competition and remained open for 1 week. The questionnaire employed a retrospective pre-post design, in which participants rated the same items at different time points (e.g., before and after the competition). All items were rated using a 5-point Likert scale (1 = very low, 2 = low, 3 = moderate, 4 = high, and 5 = very high).

The questionnaire included items assessing satisfaction with the overall competition and its components (competition rounds and experience-enhancing activities); perceived difficulty levels of the MCQ and OEQ rounds; competition-acquired knowledge; application of competition-acquired knowledge in daily life; recognition of the Department of Physiology, Faculty of Medicine Siriraj Hospital; preference levels for high school subjects, defined as participants’ self-rated level of preference for each subject, including Physics, Chemistry, Biology, English, and Mathematics; Med-Interest and SI-Interest; and self-assessed physiology knowledge, rated at 3 time points (before preparation, during preparation, and after competition), as detailed in Supplementary Table S1. All questionnaire responses were self-reported and were separate from competition scoring or adjudication. There was a 99.3% (569/573) response rate.

Furthermore, the questionnaire included 2 optional open-ended questions regarding (1) participants’ impressions of the competition and (2) suggestions. Of the 569 respondents, 303 responded to the question regarding their impressions, and 148 responded to the question regarding suggestions. Each response was reviewed and classified according to its content. Responses were classified into 4 domains: Educational impact, Participant experience, Program organization, and Overall evaluation. Within each domain, meaning units were further categorized into specific categories, as summarized in Table 3. The reported frequencies therefore represent the number of coded meaning units rather than the number of individual respondents.

The questionnaire was developed specifically for this study because no previously validated instrument directly addressed the study objectives. Item development was informed by a literature review of factors associated with motivation for studying medicine and engagement in science-related educational activities [7, 8, 11]. To ensure content validity, the questionnaire was reviewed by 4 faculty members with expertise in medical education and physiology, and their feedback was incorporated to improve item clarity, coverage of relevant constructs, and logical flow. Because the questionnaire was designed primarily to collect descriptive information rather than to establish a formal psychometric scale, and most items were analyzed individually rather than as latent multi-item constructs, exploratory or confirmatory factor analysis was not performed.

### Post-competition review

Following completion of the competition, the organizing committee conducted a departmental After Action Review (AAR) as part of routine program evaluation. Representatives from each operational team reviewed the implementation of the competition and documented strengths, areas for improvement, and recommendations. The findings were summarized descriptively (Table 4) and organized according to the same 4 evaluation domains used for participants’ open-ended responses, providing a common framework for presenting both participant and organizer perspectives.

### Subgroup analysis

In the analysis of Med-Interest, SI-Interest, and self-assessed physiology knowledge, participants’ responses were categorized based on competition phases and their preference levels for high school subjects.

Age- or grade-level subgroup analyses were not performed because the competition was designed as an academic outreach activity rather than for comparison of academic ability across grade levels.

### Statistical analysis

Data analysis was performed using IBM Statistical Package for Social Science (SPSS) Statistics version 30. Descriptive statistics were used to summarize questionnaire responses. Nonparametric tests were used because the variables were ordinal and measured using Likert scales. For comparisons of 2 related values (before-after), the Wilcoxon signed-rank test was used. Effect sizes for the pre-post comparisons of Med-Interest and SI-Interest were calculated using Cohen’s d. Change scores were calculated for each participant as the difference between after-competition and before-competition ratings and were summarized descriptively, based on paired responses provided by the same participant within the questionnaire. For comparisons of self-assessed physiology knowledge across 3 related time points within the same participants, including subgroup analyses according to preference levels for high school subjects, the Friedman test with post hoc pairwise comparisons was used. For comparisons between groups with different preference levels for high school subjects, the Kruskal-Wallis test with post hoc pairwise comparisons was used. Correlations between variables were analyzed using Spearman’s rank-order correlation coefficient (ρ). Results are presented as mean (95% confidence interval [CI]) and Spearman’s ρ (95% CI). Statistical significance was set at *P*<.05. Responses to the optional open-ended questions and the organizers’ AAR were summarized descriptively according to the 4-domain framework described above.

## Results

### Participants’ perspectives on competition activities

Participants’ perspectives on competition activities are presented as numbers, means (95% CI), and medians in Table 1.

**Table 1:** Participants’ perspectives on competition activities

Questions	Number	Mean (95% CI)	Median
Satisfaction in overall competition	569	4.5 (4.4–4.5)	5
Satisfaction in competition activities			
MCQ round management	569	4.4 (4.3–4.4)	4
MCQ round computer-based examination system	569	4.5 (4.4–4.5)	5
Written round	256	4.2 (4.1–4.3)	4
Semi-finals	220	4.2 (4.1–4.3)	4
Finals	213	4.3 (4.2–4.4)	4
Satisfaction in experience-enhancing activities			
Gross anatomy laboratory visit	504	4.3 (4.3–4.4)	5
Museum visit	454	4.3 (4.2–4.3)	5
Performance activity	432	4.3 (4.3–4.4)	5
Perceived difficulty levels			
MCQ round difficulty levels	569	3.6 (3.6–3.7)	4
OEQ round difficulty levels	230	4.0 (3.9–4.1)	4
Competition-acquired knowledge	569	4.2 (4.2–4.3)	4
Application of competition-acquired knowledge in daily life	569	4.3 (4.2–4.3)	4
Recognition of Department of Physiology			
Before competition	569	3.0 (2.9–3.1)	3
After competition	569	4.0 (4.0–4.1)***	4

*CI* Confidence Interval, *Number *Number of responses, *MCQ *Multiple-choice question, *OEQ *Open-ended question

***P<.001 compared to before competition

Participants’ satisfaction with competition activities in both academic and experience-enhancing activities was greater than 4/5 across all activities, with an overall mean score of 4.5 (4.4–4.5) (Table 1). Recognition of the Department of Physiology at our institution had a mean rating of 3.0 (2.9–3.1) before the competition and was significantly higher afterward at 4.0 (4.0–4.1) (*P*<.001) (Table 1).

### Comparisons of Med-Interest, SI-Interest, self-assessed physiology knowledge, and preference levels for high school subjects

**Fig. 1:**
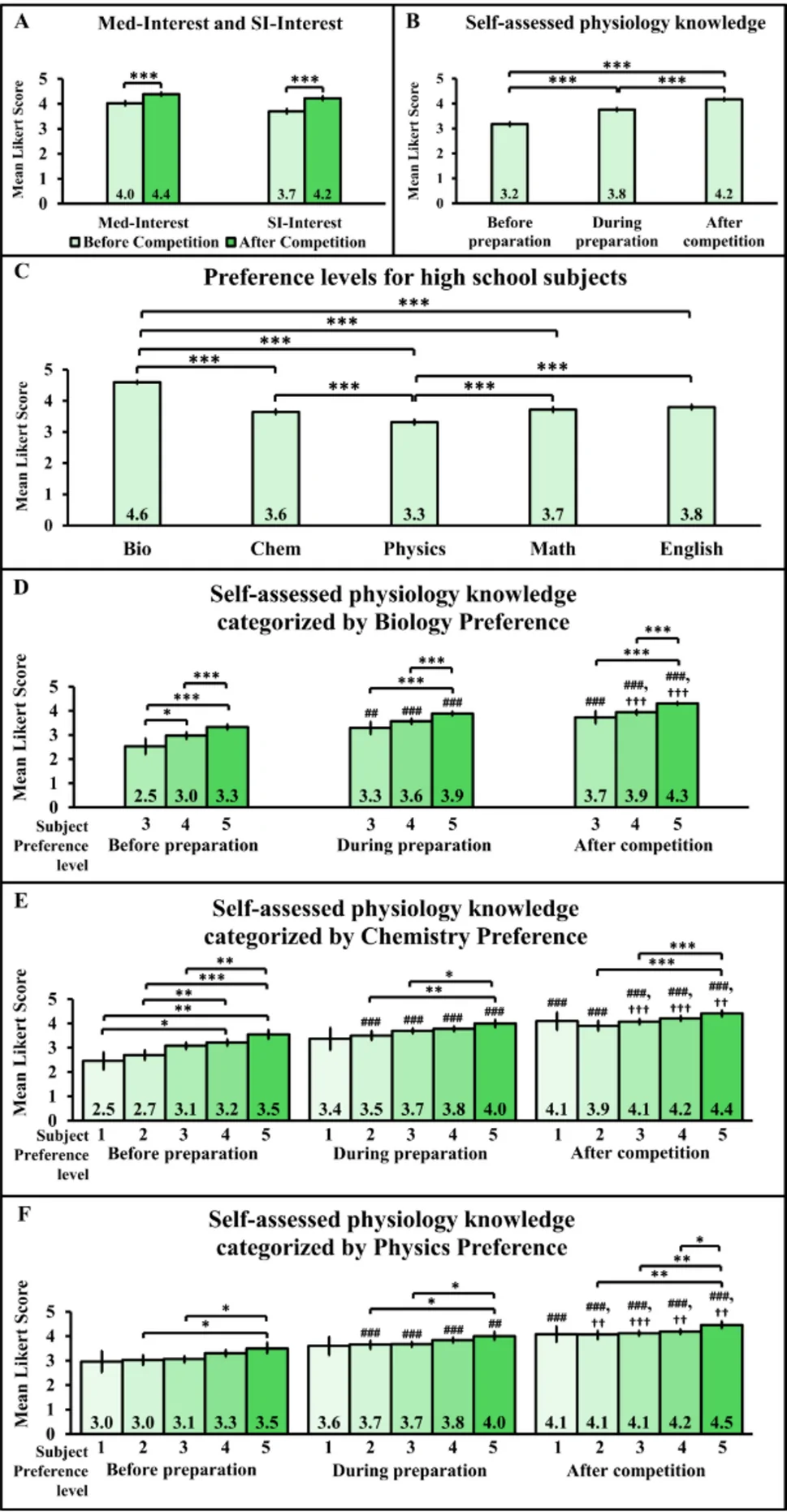
Comparisons of Med-Interest, SI-Interest, self-assessed physiology knowledge, and preference levels for high school subjects. **A** Mean Likert scores of Med-Interest and SI-Interest before and after the competition. **B** Mean Likert scores of self-assessed physiology knowledge before preparation, during preparation, and after the competition. **C** Mean preference levels for high school subjects. **D**-**F** Mean Likert scores of self-assessed physiology knowledge stratified by preference levels for Biology (**D**), Chemistry (**E**), and Physics (**F**), respectively. Med-Interest=Interest in medical school, SI-Interest=Interest in the Faculty of Medicine Siriraj Hospital, Bio=Biology, Chem=Chemistry, Math=Mathematics. In panels **A**-**C**, the y-axis represents the mean Likert scores (1 = very low, 2 = low, 3 = moderate, 4 = high, and 5 = very high), and ***P<.001 indicates comparisons between the groups connected by horizontal lines. In panels **D**-**F**, the x-axis represents participants’ preference levels for each subject (1 = very low, 2 = low, 3 = moderate, 4 = high, and 5 = very high), and the y-axis represents mean Likert scores of self-assessed physiology knowledge (1 = very low, 2 = low, 3 = moderate, 4 = high, and 5 = very high). *P<.05, **P<.01, ***P<.001 indicate comparisons between preference levels within the same time point. ^##^*P*<.01, ^###^*P*<.001 indicate comparisons with before preparation within the same preference level. ^††^*P*<.01, ^†††^*P*<.001 indicate comparisons with during preparation within the same preference level

Comparisons of Med-Interest, SI-Interest, self-assessed physiology knowledge, and preference levels for high school subjects are presented in Fig. 1.

The mean (95% CI) Med-Interest increased significantly from 4.0 (3.9–4.1) before the competition to 4.4 (4.3–4.5) after the competition, representing a relative increase of 13.8% (11.4%–16.4%) with a moderate effect size (Cohen’s d = 0.562). Similarly, SI-Interest increased from 3.7 (3.6–3.8) to 4.2 (4.1–4.3), corresponding to a 23.1% increase (19.3%–27.2%) with a moderate effect size (Cohen’s d = 0.675) (all *P*<.001) (Fig. 1A). Self-assessed physiology knowledge increased progressively from before preparation to during preparation and after the competition (all *P*<.001) (Fig. 1B). Regarding preference levels for high school subjects, Biology was rated highest and was significantly higher than all other subjects (all *P*<.001), followed by English, Mathematics, Chemistry, and Physics (Fig. 1C). Participants with higher preferences for Biology (Fig. 1D), Chemistry (Fig. 1E), and Physics (Fig. 1F) consistently reported higher levels of self-assessed physiology knowledge across all time points. Within each preference level, self-assessed physiology knowledge increased significantly from before preparation to during preparation and after the competition in most comparisons (*P*<.05) (Fig. 1D-F).

### Comparisons of Med-Interest and SI-Interest categorized by preference levels for high school subjects

**Fig. 2:**
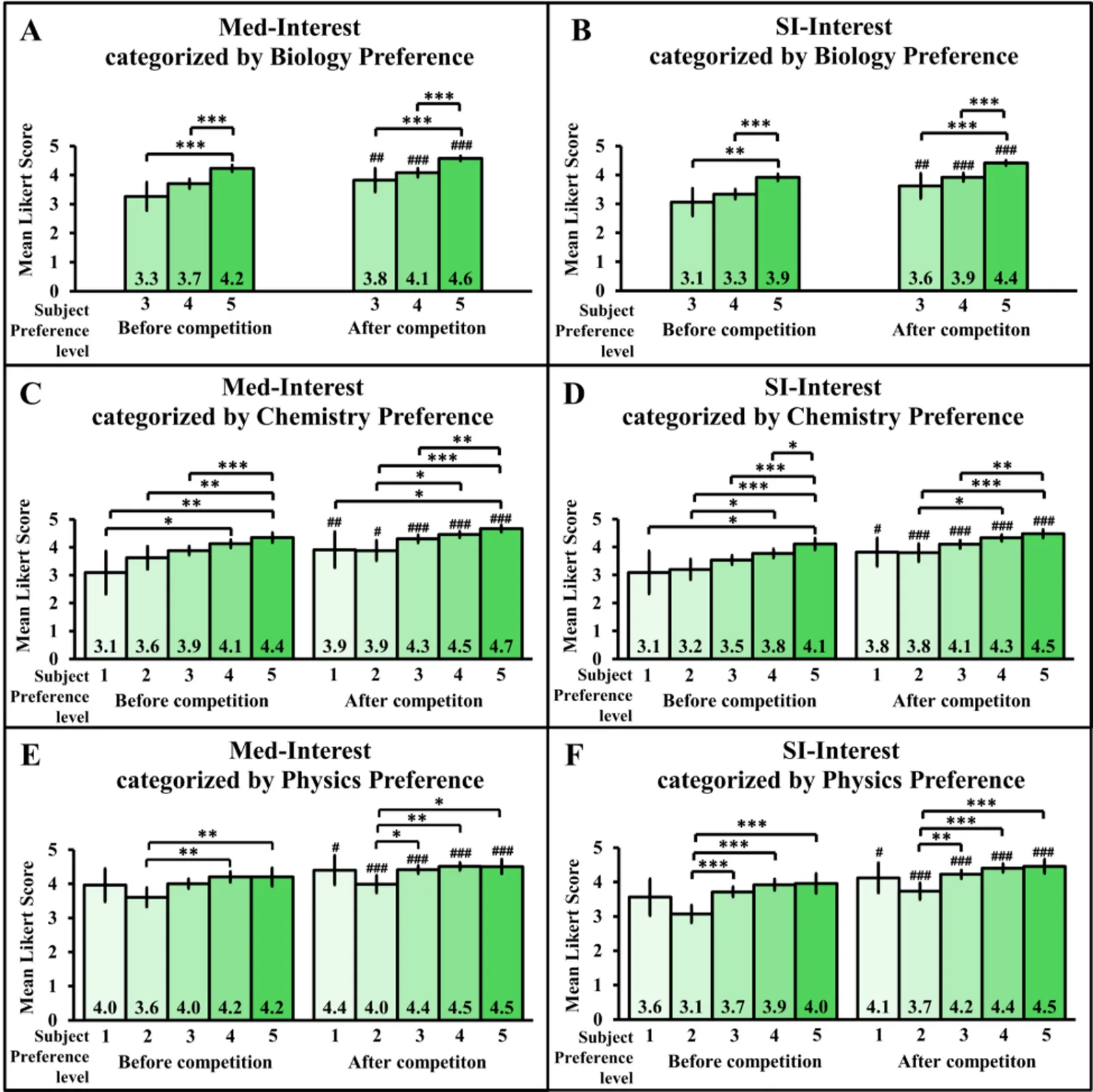
Comparisons of Med-Interest and SI-Interest categorized by preference levels for high school subjects. **A**-**B** Med-Interest and SI-Interest categorized by Biology preference levels, respectively. **C**-**D** Med-Interest and SI-Interest categorized by Chemistry preference levels, respectively. **E**-**F** Med-Interest and SI-Interest categorized by Physics preference levels, respectively. Med-Interest=Interest in medical school, SI-Interest=Interest in the Faculty of Medicine Siriraj Hospital. The x-axis indicates participants’ preference levels for each high school subject (1 = very low, 2 = low, 3 = moderate, 4 = high, and 5 = very high), and the y-axis represents the mean Likert scores for Med-Interest or SI-Interest (1 = very low, 2 = low, 3 = moderate, 4 = high, and 5 = very high). *P<.05, **P<.01, ***P<.001 compared between subject preference levels within the same time point. ^#^*P*<.05, ^##^*P*<.01, ^###^*P*<.001 compared with before competition within the same preference level

Comparisons of Med-Interest and SI-Interest categorized by preference levels for high school subjects are shown in Fig. 2.

Participants with higher preferences for Biology, Chemistry, and Physics generally reported higher levels of Med-Interest (Fig. 2A, C, and E, respectively) and SI-Interest (Fig. 2B, D, and F, respectively) throughout the competition. Among these subjects, both Med-Interest and SI-Interest significantly increased from before to after the competition for all preference levels (*P*<.05 all) (Fig. 2A–F).

### Correlations of Med-Interest and SI-Interest with other factors

Correlations of Med-Interest and SI-Interest with other factors are shown in Table 2.

**Table 2:** Correlations of Med-Interest and SI-Interest with other factors

Items	Med-Interest				SI-Interest			
	Before competition		After competition		Before competition		After competition	
	ρ (95% CI)	*P*	ρ (95% CI)	*P*	ρ (95% CI)	*P*	ρ (95% CI)	*P*
Preference in high school subjects								
Biology	0.292 (0.212–0.367)	< 0.001	0.315 (0.237–0.390)	< 0.001	0.268 (0.188–0.345)	< 0.001	0.297 (0.218–0.373)	< 0.001
Chemistry	0.216 (0.133–0.295)	< 0.001	0.217 (0.135–0.296)	< 0.001	0.237 (0.156–0.316)	< 0.001	0.214 (0.132–0.294)	< 0.001
Physics	0.161 (0.077–0.242)	< 0.001	0.123 (0.039–0.206)	0.003	0.215 (0.133–0.294)	< 0.001	0.213 (0.131–0.293)	< 0.001
Mathematics	0.122 (0.038–0.205)	0.003	0.138 (0.054–0.220)	< 0.001	0.188 (0.105–0.268)	< 0.001	0.186 (0.103–0.266)	< 0.001
English	0.129 (0.045–0.212)	0.002	0.100 (0.015–0.183)	0.017	0.081 (-0.003–0.165)	0.052	0.030 (-0.055–0.114)	0.474
Self-assessed physiology knowledge								
Before preparation	0.245 (0.164–0.323)	< 0.001	0.188 (0.105–0.268)	< 0.001	0.309 (0.231–0.384)	< 0.001	0.252 (0.171–0.329)	< 0.001
During preparation	0.241 (0.160–0.319)	< 0.001	0.230 (0.149–0.309)	< 0.001	0.244 (0.163–0.322)	< 0.001	0.242 (0.161–0.320)	< 0.001
After competition	0.232 (0.150–0.310)	< 0.001	0.290 (0.211–0.366)	< 0.001	0.205 (0.123–0.285)	< 0.001	0.261 (0.180–0.338)	< 0.001
Recognition of Department of Physiology								
Before competition	0.332 (0.255–0.405)	< 0.001			0.426 (0.354–0.493)	< 0.001		
After competition			0.296 (0.217–0.371)	< 0.001			0.340 (0.263–0.413)	< 0.001
Overall Satisfaction			0.266 (0.186–0.343)	< 0.001			0.254 (0.173–0.332)	< 0.001
Satisfaction in academic activities								
MCQ round management			0.219 (0.137–0.298)	< 0.001			0.194 (0.111–0.274)	< 0.001
MCQ round computer-based examination system			0.240 (0.158–0.318)	< 0.001			0.229 (0.147–0.307)	< 0.001
Written round			0.195 (0.070–0.313)	0.002			0.254 (0.132–0.368)	< 0.001
Semi-finals			0.317 (0.189–0.435)	< 0.001			0.282 (0.152–0.403)	< 0.001
Finals			0.315 (0.185–0.435)	< 0.001			0.268 (0.135–0.392)	< 0.001
Satisfaction in experience-enhancing activities								
Gross anatomy laboratory visit			0.229 (0.142–0.313)	< 0.001			0.280 (0.195–0.361)	< 0.001
Museum visit			0.260 (0.170–0.347)	< 0.001			0.293 (0.204–0.377)	< 0.001
Performance activity			0.252 (0.159–0.341)	< 0.001			0.246 (0.153–0.336)	< 0.001
Perceived level of quiz difficulty								
MCQ round			0.074 (-0.011–0.157)	0.080			0.135 (0.051–0.217)	0.001
OEQ rounds			0.223 (0.092–0.346)	< 0.001			0.229 (0.099–0.352)	< 0.001
Competition-acquired knowledge			0.271 (0.191–0.348)	< 0.001			0.295 (0.216–0.370)	< 0.001
Application of competition-acquired knowledge in daily life			0.321 (0.243–0.395)	< 0.001			0.303 (0.224–0.378)	< 0.001

*Med-Interest *Interest in medical school, *SI-Interest *Interest in Faculty of Medicine Siriraj Hospital, *MCQ *Multiple-choice question, *OEQ *Open-ended question, *ρ* Spearman’s rank correlation coefficient

Overall, the observed correlations were statistically significant but weak in magnitude. Among high school subjects, both Med-Interest and SI-Interest showed the strongest correlations with preference for Biology before and after the competition (Table 2).

Regarding self-assessed physiology knowledge and recognition of the Department of Physiology at our institution, both Med-Interest and SI-Interest showed positive correlations with these factors before and after the competition (Table 2).

Overall satisfaction with the competition activities was positively correlated with Med-Interest and SI-Interest after the competition. Among academic activities, the strongest correlations were observed with satisfaction in the semi-finals round, whereas among experience-enhancing activities, the strongest correlations were observed with satisfaction in the museum visit (Table 2).

In addition, Med-Interest and SI-Interest after the competition were positively correlated with perceived OEQ difficulty, competition-acquired knowledge, and application of competition-acquired knowledge in daily life (Table 2). Overall, weak but consistent positive associations were observed between Med-Interest and SI-Interest and both academic and experiential activities of the competition.

### Participants’ impressions of the competition and suggestions from the optional open-ended questionnaire responses

Participants’ impressions of the competition and suggestions from the optional open-ended questionnaire responses are summarized in Table 3.

**Table 3:** Summary of participants’ impressions of the competition and suggestions from the optional open-ended questionnaire responses

Do- main	Category	Participants’ impressions of the competition	No. of coded meaning units	Summary of participants’ suggestions	No. of coded meaning units
Educational impact	Knowledge acquisition and learning	Participants reported acquiring new knowledge, improving understanding, applying knowledge, preparing for future study, and broadening their learning experiences.	55	Participants suggested providing more opportunities for knowledge exchange among teams.	1
	Interest in physiology	Participants described increased physiology knowledge, greater interest in physiology, and motivation to further study physiology.	11		
	Interest in medicine	Participants reported increased interest in medicine, stronger intention to pursue medical school, and greater confidence in choosing medicine as a future career.	5		
	Interest in SI	Participants reported increased interest in studying at SI, greater familiarity with the faculty, improved perceptions of the learning environment, and stronger intention to pursue medical education there.	37		
	Motivation and personal growth	Participants described increased motivation for future study, inspiration for career planning, greater self-awareness, identification of strengths and weaknesses, and commitment to self-improvement.	22		
Participant experience	Competition experience	Participants positively evaluated the competition format, examination system, examination questions, and examination difficulty as appropriate, enjoyable, and intellectually challenging.	69	Participants suggested improving the competition format, increasing team-based components, refining the MCQ and semi-final rounds, improving examination difficulty, clarifying questions and answers, and improving the quiz-master procedures.	91
	Experience-enhancing activities	Participants identified SI talk, gross anatomy laboratory visit, museum visit, and performance activity as memorable experiences that enriched their understanding of medicine and exposed them to medical school life.	111	Participants suggested increasing activity time for SI talk, gross anatomy laboratory visit, museum visit, and performance activity.	39
	Experiential learning	Participants described gaining authentic experiences, exploring new environments, developing teamwork skills, and gaining valuable personal experiences beyond classroom learning.	33		
	Peer interaction and networking	Participants appreciated meeting students from different schools, making new friends, interacting with peers with similar interests, and building supportive relationships.	27	Participants suggested allowing more relaxed opportunities to interact with other participants.	1
Program organization	Organization and support	Participants praised the organization of the competition, registration process, logistics, venue and facilities, food, staff support, medical students, faculty members, and overall management.	208	Participants suggested improving overall organization, publicity, application and registration systems, late-night scheduling, livestream and audiovisual systems, facilities, food coupons, and dining areas.	49
Overall evaluation	Overall satisfaction	Participants expressed high overall satisfaction and described the competition as enjoyable, worthwhile, well organized, and an experience they hoped would continue in future years.	75	Some participants indicated that no changes were needed or that the program should remain as it was.	2

*No.* number, *SI *Faculty of Medicine Siriraj Hospital, *MCQ *Multiple-choice questions

### Organizing committee reflections

Reflections from the organizing committee obtained through the departmental AAR are summarized in Table 4.

**Table 4:** Summary of findings from the organizing committee’s After Action Review (AAR)

Do-main	Category	Strengths	Areas for improvement	Recommendations
Educational impact	Knowledge acquisition and learning	Competition content was designed within the upper-secondary school curriculum using reference books accessible to all schools. Examination questions were reviewed with experts teaching at the upper-secondary level to ensure appropriate difficulty, avoid duplication, and cover diverse physiology content, helping reinforce the importance of knowledge learned in high school.	Examination difficulty, question format, examination blueprint, answer explanation procedures, competition rules, examination logistics, and fairness across examination rounds required refinement.	Refine the examination blueprint and question design, standardize answer explanation procedures, communicate competition rules clearly before the competition, improve examination logistics, and review examination fairness across rounds.
	Interest in physiology/ medicine/ SI	SI talk, visits to the gross anatomy laboratory and museums, and the performance activity were organized to introduce participants to medical education and SI.	Communication, scheduling, live broadcasting, participant flow, and participant engagement during live-broadcast sessions could be improved.	Improve activity scheduling, communication, live-streaming systems, participant flow, and accommodate all participants in a venue where they can participate in activities in person.
	Motivation and personal growth	SI talk featured interesting speakers who inspired participants.	Q&A time were limited.	Increase time for Q&A.
Participant experience	Competition experience	Competition rules and examination formats were communicated through multiple channels before the competition and reiterated before the examination. The SELECx examination system was tested before use. Examinations started and finished on time, answer explanations were delivered efficiently, and the roles of the quizmaster and judges, together with predefined scoring rules, supported smooth competition management.	Some competition rules were not communicated clearly or sufficiently in advance. Examination instructions, consistency between login information presented in participant materials and on the examination login screen, availability of a “no answer” response option, participant support, staffing, restroom availability, and the timing of question distribution to quizmasters required improvement.	Communicate competition rules clearly before the event, standardize examination instructions, ensure consistency between participant materials and the examination login screen, include a “no answer” response option, increase staffing at examination sites, increase restroom availability, and provide examination questions to quizmasters earlier to allow sufficient preparation time.
	Experience- enhancing activities/ Experiential learning	SI talk, the gross anatomy laboratory visit, museum visits, and the performance activity were organized. Activities were appropriate for large groups, participant flow was well managed, sufficient staff were assigned to each area, and activities finished on schedule. The gross anatomy laboratory visit was supported by the Department of Anatomy, with appropriate specimens, no formalin odor, suitable viewing time, and well-organized participant flow.	Transportation, activity scheduling, waiting time, communication about locations and visiting times, live-streaming stability, microphone/audio support, and coordination between activity areas required improvement.	Improve transportation planning, consolidate activity information in the program booklet, clarify activity schedules and locations, improve live-streaming and audio systems, add activities during waiting time, and strengthen coordination with activity providers.
	Peer interaction and networking			
Program organization	Organization and support	Competition information was disseminated through the website, Facebook, email, private Facebook groups, and Dek-D TCAS. Google Forms, QR code-based registration, and clearly organized registration procedures facilitated participant management. Collaboration among multiple departments supported venue preparation, traffic management, catering, transportation, Facebook Live broadcasting, and overall event operations.	Participant registration data inaccuracies, lack of an integrated online registration system, venue signage, parking, IT and audiovisual support, manpower allocation, document processing, sponsor planning, financial procedures, and cross-team coordination required improvement.	Develop an integrated online registration system, improve signage, parking, IT and audiovisual support, clarify staff roles, improve document and financial procedures, plan sponsor acquisition earlier, and strengthen coordination among organizing teams.
Overall evaluation	Overall satisfaction	The competition attracted a large number of applicants and achieved its objectives of promoting physiology knowledge and increasing awareness of SI through nationwide participation and a combination of academic and experiential activities.	Overall planning, communication, coordination, resource estimation, contingency planning, and workload distribution required further improvement.	Maintain the program’s strengths while improving advance planning, communication, coordination, resource estimation, contingency planning, and workload distribution.

*SI* Faculty of Medicine Siriraj Hospital, *Q&A *Question and Answer, *SELECx *Siriraj e-Learning and Education Community, *TCAS *Thai University Central Admission System, *QR code* Quick Response Code, *IT *Information Technology

## Discussion

The present study assessed participants’ Med-Interest and SI-Interest before and after a physiology-based academic competition, including subgroup analyses based on preference levels for high school subjects. Participation in the competition was associated with increased Med-Interest and SI-Interest, along with increased self-assessed physiology knowledge. These increases were observed across most subject preference groups, although variations in magnitude were noted. In addition, Med-Interest and SI-Interest were positively, albeit weakly, correlated with satisfaction with competition activities, competition-acquired knowledge, and preference levels for high school subjects. To the best of our knowledge, this is the first study to compare participants’ Med-Interest before and after a physiology academic competition with subgroup analysis based on preference levels for high school subjects. Beyond this novelty, the study provides an evaluation of a competition model that integrates academic challenge, gamification, and experience-enhancing activities within a real-world educational setting.

Using Expectancy-Value Theory [9] as a conceptual framework, the findings of this study may be interpreted in relation to motivational beliefs, task value, and expectancy of success. Within the domain of motivational beliefs, particularly self-concept, participants with higher self-assessed physiology knowledge and stronger preferences for science-related subjects (Biology, Chemistry, and Physics) tended to report higher Med-Interest and SI-Interest, which may reflect differences in perceived competence or domain-specific self-concept. Biology was also the highest-rated subject overall, which may indicate a stronger biology-related self-concept, potentially contributing to greater Med-Interest. This interpretation is consistent with previous work showing that students with a positive self-concept tend to exhibit greater interest in relevant future careers. For example, those who rated their computer and machinery skills highly aspired to science-related careers [12].

Subgroup analyses further showed that participants with higher preference levels for science-related subjects also reported higher levels of self-assessed physiology knowledge, Med-Interest, and SI-Interest before the competition. Following the competition, increases in self-assessed physiology knowledge, Med-Interest, and SI-Interest were observed across nearly all preference levels in science-related subjects, suggesting that the competition experience was associated with increased perceived knowledge and interest across participants with different initial subject preferences. In addition, self-assessed physiology knowledge was positively correlated with both Med-Interest and SI-Interest. These results may indicate that participants who perceived greater gains in physiology knowledge were also more likely to report higher Med-Interest and SI-Interest. The quantitative findings were further complemented by participants’ optional open-ended responses, which described gaining new knowledge, increased interest in physiology and medicine, greater familiarity with the organizing institution, positive perceptions of its learning environment, and increased motivation for future study and career planning (Table 3). These quantitative and qualitative findings are consistent with a previous study showing that physiology presentations and activities among adolescents administered by undergraduate physiology students increased their self-reported science and physiology knowledge, as well as their interest in science, physiology, and research [13], and with previous studies demonstrating that out-of-school science competitions and extracurricular activities are associated with students’ motivation toward science-related careers [6, 11, 14]. Taken together, the present findings suggest that physiology-based outreach activities may engage students across a broad range of science interests rather than only those with a strong preference for Biology. Such information may be useful when designing outreach programs intended to engage students with diverse academic backgrounds and interests.

Perceived difficulty, particularly in the OEQ rounds, was positively associated with both Med-Interest and SI-Interest. This may reflect a perceived level of challenge that participants found engaging, in which tasks are sufficiently demanding to engage participants while remaining achievable, thereby supporting perceived competence and potentially fostering intrinsic motivation, consistent with self-determination theory [9, 15]. Such experiences may, in turn, relate to expectancy of success, as Expectancy-Value Theory proposes that perceived task difficulty is one of the motivational beliefs shaping students’ expectations for success [16].

The competition itself may also be understood as a social influence, shaping participants’ impressions and generating positive affective memories. In this context, participants’ impressions of the competition may be viewed as a cognitive appraisal through which social experiences were interpreted and subsequently linked to motivational beliefs. These affective memories were reflected in participants’ satisfaction with both the competition and the experience-enhancing activities, including SI talk, the gross anatomy laboratory visit, the museum visit, and the performance activity. Together with the knowledge acquired during the competition, these experiences were highly rated by participants and were positively correlated with Med-Interest and SI-Interest. Although these associations were generally small in magnitude, they remained statistically significant. Participants’ optional open-ended responses further highlighted educational impact, competition experience, experience-enhancing activities, experiential learning, opportunities for peer interaction and networking, and the overall organization of the competition as memorable and valuable aspects of the program. Participants also frequently described the competition as enjoyable and worthwhile, reflecting high overall satisfaction (Table 3).

In addition, the high perceived application of competition-acquired knowledge may be interpreted as conceptually related to utility value, a component of task value, indicating that participants recognized the relevance and usefulness of the knowledge gained. This interpretation was further supported by participants’ optional open-ended responses describing knowledge acquisition, broader learning experiences, and preparation for future study (Table 3). Increased recognition of the Department of Physiology, SI, was also consistent with a previous study identifying institutional resources and public relations as factors associated with grade 12 students’ decisions to pursue medical studies [8]. These observations also support the potential role of memorable real-life learning experiences in complementing academic content and fostering student motivation.

Overall, the present findings suggest that participation in the competition was associated with increased interest in medical school, particularly in the organizing institution, and may be interpreted in the context of the Expectancy-Value Theory. The greater relative increase in SI-Interest may reflect increased familiarity with the organizing institution and its learning environment. Participants’ optional open-ended responses similarly described increased familiarity with SI, positive perceptions of its learning environment, and greater interest in studying at the institution, providing contextual support for this interpretation (Table 3).

In addition to participants’ optional open-ended responses, organizers’ reflections obtained through the departmental AAR provided organizer perspectives on the competition. Whereas participants primarily emphasized educational impact, memorable learning experiences, and overall satisfaction, organizers focused on program implementation, identifying both operational strengths and opportunities for future improvement. The integration of participant and organizer perspectives across the 4 evaluation domains strengthened the overall program evaluation by incorporating perspectives from multiple stakeholders.

Following the departmental AAR, the evaluation findings, including the competition results, the numbers of participating schools and students, and a summary of participants’ questionnaire findings on their impressions and suggestions, were presented at the Faculty Committee Meeting, where several committee members expressed strong appreciation for the program and its positive outcomes. Furthermore, information describing the competition, including participation statistics, major competition activities, and the competition results, including the award-winning teams, was subsequently published on the Faculty website in both Thai [17] and English [18] and was also incorporated into Faculty reports and presentations.

As a real-world outreach activity rather than a controlled intervention, this competition may offer practical insights into how academic challenge, gamification, and experiential learning can be integrated to enhance student engagement and interest. These findings, together with participants’ optional open-ended responses, may help inform the design of future medical science outreach and pre-medical enrichment initiatives in authentic educational settings.

This study has several limitations. First, because this study employed a single-group retrospective pre-post design without a comparison group, causal relationships between participation in the competition and the observed changes in Med-Interest, SI-Interest, and self-assessed physiology knowledge cannot be established. Second, because pre-competition perceptions were assessed retrospectively within a single post-competition survey, recall bias may have influenced the reported before-and-after comparisons. Furthermore, Med-Interest, SI-Interest, and self-assessed physiology knowledge were measured using self-reported Likert-scale ratings and therefore reflect participants’ perceptions rather than objective measures. Third, participants were selected by their schools and may not be representative of all high school students. Students who chose to participate in a science-based academic competition may have had a higher baseline interest in science or medicine, introducing potential self-selection bias. Fourth, although all participants were able to complete the educational and experience-enhancing activities and could observe the later competition rounds either onsite or online, only a subset of participants advanced to the Qualifying, Semi-final, and Final rounds. Therefore, participants’ emotional engagement and competition experiences may have differed according to the stage reached in the competition, which may have influenced their post-competition perceptions and questionnaire responses. Fifth, because the questionnaire was administered by the organizing institution, participants’ ratings of SI-Interest may have been influenced by social desirability bias, potentially resulting in more favorable ratings of the institution. Sixth, the questionnaire was specifically developed for this study rather than based on an established validated instrument; therefore, some constructs within the Expectancy-Value Theory framework may not have been fully captured, particularly expectancy- and task value-related constructs. Seventh, as this study was conducted in a single competition organized by one institution, the generalizability of the findings to other competition formats, institutions, or cultural contexts may be limited.

In conclusion, participation in the competition was associated with increased Med-Interest, particularly SI-Interest, along with improved self-assessed physiology knowledge. Higher preference levels for Biology, Chemistry, and Physics were also associated with higher levels of both Med-Interest and SI-Interest. In addition, Med-Interest and SI-Interest showed weak but statistically significant positive correlations with self-assessed physiology knowledge and academic and experiential aspects of the competition. These findings support the potential relevance of an innovative competition model that integrates academic challenge, gamification, and experiential learning within a real-world educational setting. Such an approach may offer practical insights for designing engaging and educationally meaningful medical science outreach and pre-medical enrichment initiatives, offering guidance for the development of programs that foster student interest in medical education. These quantitative findings were further supported by participants’ optional open-ended responses describing knowledge acquisition, increased interest in medicine, greater familiarity with the organizing institution, and increased motivation for future study. Future research should consider the use of established motivational assessment tools, such as the Expectancy-Value-Cost (EVC) Survey, and longitudinal studies tracking students’ educational pathways and decisions related to medical school may provide further insight into the longer-term impact of such competitions.

**Fig. 3:**
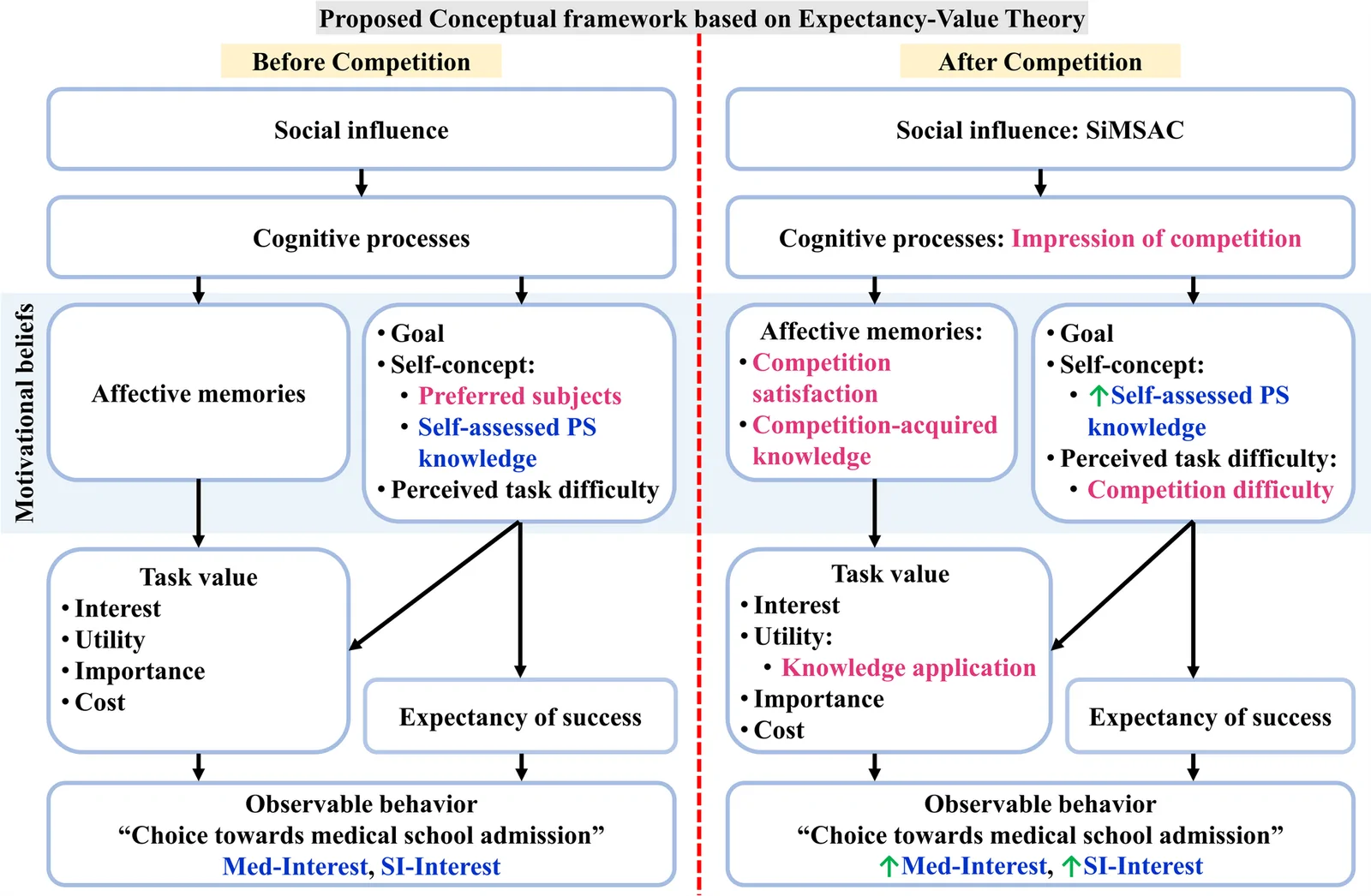
Proposed conceptual framework based on Expectancy-Value Theory illustrating the potential relationships suggested by the study findings. Blue text indicates variables assessed using retrospective pre- and post-competition ratings, whereas pink text indicates variables assessed at a single post-competition time point. Other elements are included based on the Expectancy-Value Theory framework to support interpretation of the observed relationships SiMSAC = The 1^st^ Siriraj Medical Sciences Academic Competition (SiMSAC), themed ‘Physiology’, PS = Physiology, Med-Interest = Interest in Medical School, SI-Interest = Interest in the Faculty of Medicine Siriraj Hospital

A proposed conceptual framework based on Expectancy-Value Theory illustrating the potential relationships suggested by the study findings is shown in Fig. 3.

The framework summarizes how both academic and experiential components of the competition may have contributed to students’ motivation toward medical studies. 

## Supplementary Information

Supplementary Material 1.

## Data Availability

The datasets used and/or analysed during the current study are available from the corresponding author on reasonable request.
