# Supplementary material for: Changes in high school students’ interest in medical school following a physiology-based medical science competition: a questionnaire-based study
Source: BMC Med Educ. 2026 Aug 3;26:1750. doi: 10.1186/s12909-026-10081-5 (PMC13721529; doi:10.1186/s12909-026-10081-5)
Supplement: Supplementary file 1 [file 12909_2026_10081_MOESM1_ESM.docx]

**Supplementary**

**Supplementary Table S1:**

**Satisfaction Questionnaire on the Organization of the 1st Siriraj Medical Sciences Academic Competition (SiMSAC)**

Please respond to the following questions using the following scale: 1=very low, 2=low, 3=moderate, 4=high, and 5=very high

**Questions**

**Satisfaction with overall competition**

**Satisfaction with competition activities**

- MCQ round management
- MCQ round computer-based examination system
- Written round
- Semi-finals
- Finals

**Satisfaction with experience-enhancing activities**

- Gross anatomy laboratory visit
- Museum visit
- Performance activity

**Perceived difficulty levels**

- MCQ round difficulty levels
- OEQ round difficulty levels

**Competition-acquired knowledge**

**Application of competition-acquired knowledge in daily life**

**Recognition of Department of Physiology, Faculty of Medicine Siriraj Hospital**

- Before competition
- After competition

**Preference levels for high school subject**

- Physics
- Chemistry
- Biology
- English
- Mathematics

**Interest levels toward medical school (Med-Interest)**

- Before competition
- After competition

**Interest levels toward Faculty of Medicine Siriraj Hospital (SI-Interest)**

- Before competition
- After competition

**Self-assessed knowledge**

- Before preparation
- During preparation
- After competition

**Impressions of the competition** (Optional)

**Suggestions** (Optional)

**Supplementary Appendix 1:** A detailed description of the competition format and activities

For the academic component, the competition consisted of 4 rounds:

1. MCQ round: Each participant answered 90 questions individually, with 1 point awarded for each correct answer and 0.25 points deducted for each incorrect answer. The questions focused on the physiology topics covered in Biology textbooks (Volumes 4 and 5) used in the Additional Science and Technology (Biology) course, aligned with the Science Learning Area standards (revised 2017) under the Basic Education Core Curriculum (2008).

2. Qualifying round: The questions were based on physiology topics covered in the Biology textbooks described above. Each question allowed 15 seconds for teams to write their answers. A quiz master presented the questions and verified the answers using predetermined answer keys, and 3 physician faculty members served as judges in each round.

3. Semi-finals: Each oral OEQ round consisted of 10 questions. Teams were given up to 30 seconds to respond to each question. The questions were presented by a quiz master using predetermined key answers, and responses were evaluated by 3 physician faculty members serving as judges in each round. Each team selected questions randomly, earning 3 points for a correct answer. If the answer was incorrect, the team received 0 points, and the remaining teams had the opportunity to steal the right to answer by pressing a buzzer, with the fastest team earning the opportunity to respond, with each correct response earning 2 or 1 point, respectively, and 1 point deducted for an incorrect response.

4. Final round: A total of 4 teams competed in 12 oral OEQs using the same scoring criteria as in the Semi-finals. Each question allowed up to 45 seconds for teams to respond. A quiz master presented the questions using predetermined key answers, and 3 physician faculty members served as judges for scoring and adjudication.
